# Mitochondria-Associated Endoplasmic Reticulum Membranes in Cardiovascular Diseases

**DOI:** 10.3389/fcell.2020.604240

**Published:** 2020-11-09

**Authors:** Peng Gao, Zhencheng Yan, Zhiming Zhu

**Affiliations:** Department of Hypertension and Endocrinology, Chongqing Institute of Hypertension, Daping Hospital, Army Medical University, Chongqing, China

**Keywords:** mitochondria-associated ER membrane, cardiovascular diseases, SR-mitochondrial contact, mitochondrial bioenergetics, metabolic transition

## Abstract

The endoplasmic reticulum (ER) and mitochondria are physically connected to form dedicated structural domains known as mitochondria-associated ER membranes (MAMs), which participate in fundamental biological processes, including lipid and calcium (Ca^2+^) homeostasis, mitochondrial dynamics and other related cellular behaviors such as autophagy, ER stress, inflammation and apoptosis. Many studies have proved the importance of MAMs in maintaining the normal function of both organelles, and the abnormal amount, structure or function of MAMs is related to the occurrence of cardiovascular diseases. Here, we review the knowledge regarding the components of MAMs according to their different functions and the specific roles of MAMs in cardiovascular physiology and pathophysiology, focusing on some highly prevalent cardiovascular diseases, including ischemia-reperfusion, diabetic cardiomyopathy, heart failure, pulmonary arterial hypertension and systemic vascular diseases. Finally, we summarize the possible mechanisms of MAM in cardiovascular diseases and put forward some obstacles in the understanding of MAM function we may encounter.

## Introduction

As the main energy-producing organelles in eukaryotic cells, mitochondria are very important in maintaining the metabolism and function of cells, especially those with high energy demand, such as continuously contracting cardiomyocytes. The main energy substrate, fatty acids, or other substrates (mainly glucose, lactic acid, ketone body, etc.), require mitochondrial oxidative phosphorylation to generate enough ATP for maintaining contraction (Bertero and Maack, 2018). Mitochondrial disorders, accompanied by repressed mitochondrial oxidative activity, are detrimental to cardiac function, leading to cardiac hypertrophy and eventually HF (Buchwald et al., 1990; Bertero and Maack, 2018). On the other hand, as the main function of blood vessels is to transport oxygen to other tissues, the basal oxygen consumption of vascular ECs and smooth muscle cells is very low and the energy required is mainly from glycolysis, with concomitant lower mitochondrial content (Paul, 1983; Culic et al., 1997). However, impaired vascular mitochondrial function has been associated with metabolic transition of vascular cells and increased ROS production, resulting in the loss of EC barrier function and the increase of VSMC *trans*-differentiation and proliferation, hallmarks of arterial stiffness, lipid deposition, vascular remodeling and other vascular pathological changes (Dromparis and Michelakis, 2013). Taken together, abnormal mitochondrial metabolism plays a critical role in the development of cardiovascular diseases.

Similar as other membrane organelles, mitochondria are highly dynamic structures, and their mass, morphology, location and composition are constantly changing according to cellular requirements (Pernas and Scorrano, 2016). In addition, mounting evidence have proved the existence of physical and functional communication between mitochondria and other organelles, such as plasma membrane, nucleus and ER (Lackner, 2019). ER-mitochondrial coupling is the first and best described type of organelle interaction. The first evidence of physical interaction between these membranes comes from electron microscopy studies more than 60 years ago (Copeland and Dalton, 1959). About 30 years later, ER-mitochondrial contact was isolated by subcellular isolation using Percoll density gradient and lead to the production of the term MAMs (Vance, 1990). This contact is considered to be critically important for mitochondrial metabolism as at these sites calcium (Ca^2+^) is directly transferred from the ER storage area to the mitochondria, and increases the activities of Krebs cycle and electron transport chain, thus stimulating ATP synthesis in the mitochondrial matrix (Cardenas et al., 2010). However, overactivation of electron transport chain by excessive mitochondrial Ca^2+^, which is evident during ischemia–reperfusion, is directly linked to enhanced ROS production (Brookes et al., 2004), and mitochondrial Ca^2+^ overload also induces apoptosis by opening the mPTP on the mitochondrial outer membrane, leading to rapid collapse of the membrane potential and swelling of mitochondria (Kwong and Molkentin, 2015). Therefore, the aberrant formation of MAMs is a main step of mitochondrial dysfunction.

In addition to acting as a regulator of mitochondrial energy production, MAMs are also important in regulating the contractile function of arteries and hearts by modulating transient intracellular Ca^2+^ concentration (Herring et al., 2006; Orchard and Brette, 2008). The disorder of mitochondrial Ca^2+^ buffering mediated by MAMs often leads to the abnormal increase of Ca^2+^ in the cytoplasm, thus activating the calcium signaling pathway related to cardiac hypertrophy and HF (Frey et al., 2000). Therefore, in this review, we will first introduce the molecular components of MAMs and the evidence of ER-mitochondria communication in cardiovascular system according to their different roles in regulating mitochondrial morphology and function. We then discuss the role of MAMs in cardiac and vascular function, and how dysfunction of MAMs is associated with highly prevalent cardiovascular diseases such as HF, myocardial hypertrophy and hypertension.

## The Components and Function of Mams

### Lipid Synthesis and Transfer

Phospholipid transport and synthesis is the first recognized function of the ER mitochondrial interface (Rusinol et al., 1994). In fact, the first proteins found on the MAMs, PEMT2 and phosphatidylserine synthase 1 and 2 (PSS1/2) (Cui et al., 1993; Stone and Vance, 2000), are related to lipid metabolism, and FACL4, which is involved in the synthesis of triacylglycerol, is currently regarded as one of the most reliable MAM markers (Rusinol et al., 1994). The enrichment of cholesterol and sphingolipid in MAM is related to the accumulation of caveolin-1 (Sala-Vila et al., 2016). After synthesis, caveolin-1 is inserted into the ER, not only participates in cholesterol transport to the plasma membrane, but also regulates ER-mitochondrial cholesterol transfer (Quest et al., 2008). In addition, the enrichment of synthetic enzymes at MAM promote the local generation of main structural component of biological membranes, phosphatidylcholine, PE, and phosphatidylserine (Vance, 1990). Phosphatidylserine synthesized in ER requires mitochondrial specific phospholipase to produce PE, which is then converted to phosphatidylcholine in the ER (Shiao et al., 1995). This transferring process is carried out by ORP5 and ORP8, two proteins known to be involved in the phosphatidylserine transfer from the ER to plasma membrane or MAM (Galmes et al., 2016). In addition, phospholipid acids are synthesized in ER and must be transferred to mitochondria for modification to produce mitochondrial cardiolipin that exerts cardioprotective function (Dennis and Kennedy, 1972; Osman et al., 2011). Cardiolipin interacts strongly with, and is required for the stability and activity of many integral membrane proteins of the IMM (Musatov and Sedlak, 2017), including the mitochondrial Ca^2+^ uniporter (MCU) that mediates Ca^2+^ uptake in mitochondrial matrix (Ghosh et al., 2020). Levels of individual species of cholesterol esters, PEs, and triacylglycerols are associated with cardiovascular diseases (Stegemann et al., 2014).

### Ca^2+^ Transfer

The energy released by electron transport from the mitochondrial oxidative respiratory chain is used to form a proton gradient across the inner membrane of mitochondria, which drives ATP synthesis and also creates a driving force for Ca^2+^ absorption (Giorgi et al., 2018). However, the entry of Ca^2+^ into mitochondrial matrix is a process that exhausts mitochondrial potential and competes with ATP generation, thus requires precise regulation (Giorgi et al., 2018). Importantly, mitochondria must be exposed to high concentration of Ca^2+^ in order to take up Ca^2+^ due to the limitation of MCU on IMM (Rizzuto et al., 1998). Thus, mitochondrial Ca^2+^ uptake is most likely to occur near the Ca^2+^ releasing stores, such as ER (Giorgi et al., 2018). Through MAMs, Ca^2+^ is transferred directly from the ER to mitochondria and controls key mitochondrial functions, such as apoptosis and energy generation (Giorgi et al., 2018). This local and rapid uptake of mitochondrial Ca^2+^ can prevent excessive increase of cytosolic Ca^2+^ and control the Ca^2+^ signals to occur locally (Laude and Simpson, 2009).

The effective transfer of Ca^2+^ between the ER and mitochondria is mediated by a complex of multiple proteins. The main channel of Ca^2+^ release of the ER, Inositol-1,4,5-triphosphate receptor type 1 (IP3R1), is responsible for forming a high Ca^2+^ domain in the ER vicinity. The VDAC1 acts as a Ca^2+^ uptake channel in the OMM. The third component of the complex is mitochondrial stress 70 protein, also known as GRP75, which connects two channels through their cytosolic portions to form VDAC1/GRP75/IP3R1 channel complex (Szabadkai et al., 2006). The overexpression of GRP75 does not increase the ER-mitochondrial contact, so GRP75 may play a role in the established contact points (Szabadkai et al., 2006). In this way, Ca^2+^ is transferred directly from ER to cytosol and across the OMM, then Ca^2+^ is transported into mitochondrial matrix via MCU (Baughman et al., 2011; De Stefani et al., 2011). In excitable cell types, such as cardiomyocytes and VSMCs, the RYR on SR is also present in MAMs and plays a key role in organelle Ca^2+^ transfer in these cells (Eisner et al., 2013).

Recently, a new family of TRPM8 channel isoforms as functional ER Ca^2+^ release channels expressed in MAMs has been identified (Bidaux et al., 2018). We also confirmed that SR-resident TRPM8 participated in the regulation of cellular and mitochondrial Ca^2+^ homeostasis in the VSMCs. TRPM8 activation by menthol antagonized angiotensin II (AngII)-induced mitochondrial respiratory dysfunction and excess ROS generation by preserving mitochondrial Ca^2+^-dependent PDH activity, thus lowered blood pressure in cold or in AngII-induced hypertensive mice (Xiong et al., 2017). Meanwhile, restoration of ER-mitochondrial Ca^2+^ transfer by activation of TRPM8 seems to be beneficial for restricting cytosolic Ca^2+^ signaling that accounts for vascular constriction (Sun et al., 2014). However, whether there are other TRP channels located in MAM and involved in the Ca^2+^ transfer from ER to mitochondria remains to be further investigated.

### Mitochondrial Dynamics

Mitochondria are dynamic organelles continuously undergoing fusion and fission. A proper balance between these two opposing processes is essential for cell survival and for maintaining the shape, the size and the number of mitochondria (Hoppins and Nunnari, 2012; Youle and van der Bliek, 2012). The main mitochondrial dynamic protein accounting for fission is DRP1. It is a cytosolic GTPase, which is recruited from the cytoplasm to form a contractile ring on the mitochondria, thus driving the cleavage process. Mitochondrial fission often occurs at positions where ER tubules contact and constrict mitochondria and facilitates the recruitment of DRP1 (Friedman et al., 2011). On the other hand, the process of mitochondrial fusion is controlled by GTPases mitofusin 1 (MFN1), mitofusin 2 (MFN2) and mitochondrial dynamic-like 120 kDa protein [also known as optic atrophy protein 1 (OPA1)]. As GTPases, MFN2 on the ER surface can form dimers with either MFN1 or MFN2 located on the mitochondria, and drives the fusion of outer membrane (OMM) of mitochondria (Chen et al., 2003; de Brito and Scorrano, 2008). This interaction not only determines the distance between organelles, but also enable coordinated regulation of ER and mitochondria dynamics (Chen et al., 2003; de Brito and Scorrano, 2008). However, this widely accepted model has been challenged by results from quantitative EM analysis, which demonstrate that MFN2 ablation or silencing increases the close contacts between the two organelles and facilitates Ca^2+^ transfer from the ER to mitochondria (Cosson et al., 2012; Filadi et al., 2015). Thus, MFN2 is more like a tethered antagonist, which prevents excessive proximity between the two organelles. The exact role of MFN2 in ER-mitochondria contact remains debated. OPA1 exists in the mitochondrial inner membrane (IMM) and the intramitochondrial space, which is responsible for maintaining cristae structure and mediating IMM fusion (Frezza et al., 2006; Meeusen et al., 2006).

### Autophagy, ER Stress, Inflammation and Apoptosis

In addition to regulating the morphology and function of mitochondria, MAMs are also involved in many important cellular behaviors, such as autophagy, ER stress, inflammation and apoptosis. MAMs not only provide an appropriate space for the occurrence of cell pathways, but also recruit some key regulatory factors responsible for these behaviors.

Autophagy is an evolutionarily conserved self-digestion process of intracellular material turnover in eukaryotes, which involves the formation of double-membrane vesicles called autophagosomes. The formation of autophagosome is initiated by the recruitment of pre-autophagosome marker ATG14L at the MAMs (Hamasaki et al., 2013). At rest, syntaxin-17 binds to DRP1, but in the absence of nutrients, DRP1 is replaced by ATG14L, which promotes the enrichment of different proteins involved in autophagy in MAMs (Arasaki et al., 2015). mTORC2, a key inducer of autophagy, is located in MAMs and regulates its integrity (Colombi et al., 2011). It is also required for normal cardiac physiology and ensures cardiomyocyte survival in response to pressure overload (Sciarretta et al., 2018). Growth factors stimulate the activation of mTORC2 and AKT, which are then translocated to MAMs to phosphorylate some key components of MAM maintaining mitochondrial potential, ATP production and Ca^2+^ uptake (Betz et al., 2013).

The changes of ER oxidation can lead to the aberrant formation of disulfide bonds and the accumulation of peptides, thus activating a series of intracellular reactions called UPR (Zeeshan et al., 2016). UPR as an acute response has been found in many types of cardiovascular disease (Zhang et al., 2019). The stimulation of UPR leads to three main response mechanisms: inositol-requiring enzyme 1α (IRE1α), PERK and ATF6, which regulate the protein folding ability of ER (Song et al., 2018). In the early stage of UPR, the increase of ER mitochondrial contact sites is beneficial (Bravo et al., 2011). Verfaillie et al. (2012) reported that without PERK, endogenous apoptosis induced by ER stress was weakened due to the reduction of MAM formation and hampered ROS signal transmission to adjacent mitochondria. The presence of IRE1 in MAMs determines the effectiveness of IP3R, which is conducive to the transfer of Ca^2+^ to mitochondria (Carreras-Sureda et al., 2019). These mechanisms connect ER stress and mitochondrial function, thus affecting the fate of cells. MAMs also provide a place for ROS generation. Oxidative condition activated PKCβ induces ser36 phosphorylation of p66Shc, resulting in p66Shc transfer to mitochondria or MAMs, where ROS could be produced (Giorgio et al., 2005; Pinton et al., 2007). ROS generation from p66Shc might facilitate short-term repair response (Akhmedov et al., 2015), but contribute to the development of many types of cardiovascular disease in a long term (Boengler et al., 2019).

A class of nucleotide oligomerization domain-like receptors (NLRs) sense abnormal cytosolic changes, such as microbial invasion, tissue damage and cell stress, and form multiprotein complexes called “inflammasome,” which are linked to the pathogenesis of several cardiovascular diseases (Liu et al., 2018). The NLRP3 inflammasome initiates proteolysis of pro-inflammatory cytokine interleukin 1β (IL-1β) (Gross et al., 2011). In resting state, NLRP3 localizes in cytoplasm and ER. Upon stimulation, NLRP3 inflammasome could be recruited to the MAM sites accompanied with its adaptor ASC, suggesting that NLRP3 strategically accumulates at mitochondria to sense mitochondrial damage (Zhou et al., 2011). Thus, MAMs play a critical role in initiating inflammation by acting as an inflammatory platform.

Ca^2+^ transfer from ER to mitochondria is a key factor in a series of events leading to apoptosis, and there are many proteins that control death and survival in MAMs. For example, BCL-2 protein family includes anti apoptotic and pro-apoptotic members, which control the sensitivity of cells to apoptosis signals. BCL-XL (also known as Bcl-2-like protein 1), a member of the anti-apoptotic family, partially localizes to MAM, increases Ca^2+^ transfer from ER to mitochondria as an adaptive response to increase mitochondrial bioenergetics and prevent intracellular Ca^2+^ overload after thapsigargin stimulation (Williams et al., 2016). A typical tumor suppressor PTEN has also been shown to be present in MAM. It antagonizes AKT signal, interacts with IP3R1, and enhances the transfer of Ca^2+^ from ER to mitochondria, which makes cells more sensitive to apoptosis (Bononi et al., 2013). In the process of apoptosis, extracellular signals trigger cell death by activating caspase 8, which then activates downstream caspases to decompose cells (Wu et al., 2014). A caspase 8 inhibitor located at MAM, cFLIPL, prevents caspase 8-mediated NOGO B cleavage, thus maintaining the integrity of ER morphology and ER-mitochondrial contacts (Marini et al., 2015). In conclusion, these findings suggest that apoptosis is strictly regulated in MAMs.

**FIGURE 1:**
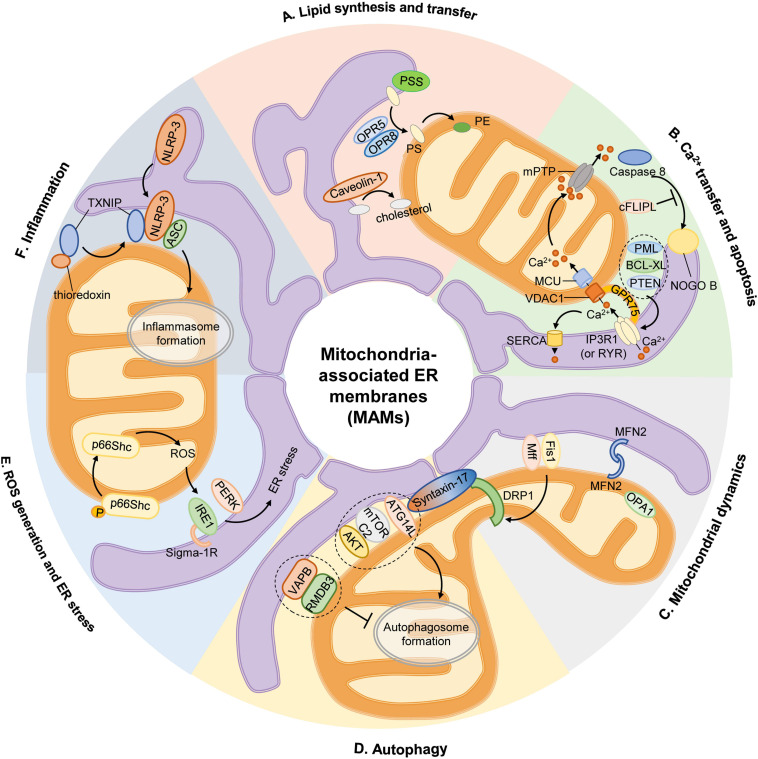
Mitochondria-associated ER membranes and cell physiology. (A) Lipid synthesis and transfer. The MAMs account for PS generation through PSS, and PS synthesized in ER is then transferred to mitochondria by ORP5 and ORP8 for further conversion to PE. In addition, caveolin-1 inserted into the ER participates in ER-mitochondrial cholesterol transfer. (B) Ca^2+^ transfer and apoptosis. Ca^2+^ transfer from ER to mitochondria is mediated by a protein complex consisting IP3R1 in ER or RYR in SR, GRP75, and VDAC1 in OMM, then Ca^2+^ is transported into mitochondrial matrix via MCU. Excessive mitochondrial Ca^2+^ uptake triggers opening of mPTP to initiate apoptosis. Activation of caspase 8 activates downstream caspases and induces NOGO B cleavage, which is inhibited by cFLIPL. Some proteins are located on MAM govern the apoptotic pathway by preserving intraorganellar Ca^2+^ transfer, such as PML, BCL-XL, or PTEN. (C) Mitochondrial dynamics. The recruitment of main mitochondrial fission protein DRP1 to MAM is regulated by Mff, Fis1 and Syntaxin-17. And mitochondrial fusion is controlled by MFN2 on OMM and OPA1 on IMM. (D) Autophagy. At rest, Syntaxin-17 binds to DRP1, but in the absence of nutrients, DRP1 is replaced by pre-autophagosome marker ATG14L, which promotes the enrichment of mTORC2 and AKT to initiate the formation of autophagosome. The anchor sets formed by VAPB in ER and RMDB3 on OMM regulate autophagy by maintaining MAMs. (E) ROS generation and ER stress. Oxidative condition induces ser36 phosphorylation of p66Shc, resulting in p66Shc transfer to MAMs and produce ROS, which stimulates ER stress via IRE1 and PERK. Sigma-1R located on MAMs could stabilize IRE1. (F) Inflammation. Upon stimulation, NLRP3 is transferred from ER to MAMs where it interacts with its adaptor ASC and TXNIP to initiate inflammasome formation. AKT, serine/threonine kinase; ASC, apoptosis-associated speck-like protein; ATG14L, autophagy-related 14-like; BCL-XL, B cell lymphoma extra-large; cFLIPL, FADD-like apoptosis regulators; DRP1, dynamin-related protein 1; ER, endoplasmic reticulum; Fis1, fission 1 protein; GRP75, chaperone 75 kDa glucose-regulated protein; IP3R1, inositol-1,4,5-triphosphate receptor type 1; IMM, inner mitochondrial membrane; IRE1, inositol-requiring enzyme 1; MAM, mitochondria-associated ER membrane; Mff, mitochondrion fission factor; MCU, mitochondrial calcium uniporter; MFN2, mitofusin 2; mTORC2, mammalian target of rapamycin complex 2; NOGO B, neurite outgrowth inhibitor B; NLRP3, pyrin domain-containing 3 protein; OPA1, optic atrophy protein 1; ORP5, oxysterol-binding protein-related protein 5; OMM, outer mitochondrial membrane; PE, phosphatidylethanolamine; PERK, protein kinase-like ER kinase; PML, promyelocytic leukemia protein; PS, phosphatidylserine; PSS, PS synthase; PTEN, phosphatase and tensin homolog; RMDB3, regulator of microtubule dynamics 3; ROS, reactive oxygen species; RYR, ryanodine receptor; TXNIP, thioredoxin interacting protein; VAMP, vesicle associated membrane protein; VAPB, VAMP-associated protein B; VDAC1, voltage-dependent anion-selective channel protein 1.

The above-mentioned functions of MAMs in cell physiology are depicted in Figure 1.

## The Role of Mams in the Regulation of Cardiovascular Physiological Function

Although MAMs were first observed more than 60 years ago, their potential role in the cardiovascular system has not been the focus of debate until the last 20 years. Significant differences in SR-mitochondrial communication were observed in cardiac and vascular tissues, which were mainly based on the cellular function regulated by this coupling. However, Ca^2+^ transfer between organelles seems to play an important role in both the heart and the vascular system.

### Heart

SR is a membrane-bound structure existing in muscle cells (myocardium and skeletal muscle), similar to ER in other cells. The main function of SR is to store Ca^2+^ (Eisner et al., 2013). In the early 1990s, different research groups provided evidence that mitochondrial Ca^2 +^ uptake was involved in myocardial contractile regulation. SR releases Ca^2+^ in response to electrical stimulation or pharmacological activation of RYR and increases mitochondrial Ca^2+^ level (Bassani et al., 1992; Negretti et al., 1993; Szalai et al., 2000). When the mitochondrial membrane potential was partially inhibited, the kinetics of cytosolic Ca^2+^ during contraction induced by electricity or caffeine was slightly changed, but the shortening degree of cardiomyocytes was reduced by more than twice, suggesting that the transfer of Ca^2+^ from SR to mitochondria was involved in cardiac contraction (Bassani et al., 1992; Negretti et al., 1993). Subsequently, studies showed that the mitochondrial Ca^2+^ oscillates synchronously with cytosolic Ca^2+^ in cardiomyocytes (Robert et al., 2001). In fact, the basal Ca^2+^ concentration in mitochondria fluctuated between 145 and 175 nmol/l in response to SR-derived Ca^2+^ release during electrical stimulation (Lu X. et al., 2013). Consistent with these observations, an increase or decrease in the level of MCU results in a lower or higher amplitude of the spontaneous cytoplasmic Ca^2+^ peak, respectively, indicating a two-way communication between these intercellular compartments (Drago et al., 2012). It is worth noting that the specific effect of mitochondrial Ca^2+^ treatment on the cytosolic Ca^2+^ level of cardiomyocytes is still controversial, which is mainly due to the moderate Ca^2+^ uptake capacity of mitochondria compared with other Ca^2+^-scavenging entities. Comparative studies showed that, despite the same biophysical properties as other tissues, MCU-derived current densities were unexpectedly lower in mouse cardiac mitochondria (Fieni et al., 2012). In addition, it is estimated that 1–15% of Ca^2+^ in the cytosol is removed by mitochondria during heart beating, while the remaining percentage is mobilized by SERCA and NCLX (Drago et al., 2012; Fieni et al., 2012). Mitochondria transiently take up Ca^2+^ and can contribute to buffering cytosolic Ca^2+^ rises, but in most cases, mitochondrial Ca^2+^ is released quite quickly through the efflux machinery, such as NCLX in cardiomyocytes. Deletion of NCLX in adult mouse hearts causes sudden death with severe myocardial dysfunction and fulminant HF due to mitochondrial Ca^2+^ overload, whereas overexpression of NCLX in the mouse heart displays potent beneficial effect of augmenting mitochondrial Ca^2+^ clearance, and protecting against ischemia-induced cardiomyocyte necrosis and HF (Luongo et al., 2017).

The Ca^2+^ transfer from SR to mitochondria is mainly restricted in the MAM regions, where local high Ca^2+^ domains are formed. The distance between SR, RYR and OMM is nearly 40 nm and the concentration of Ca^2+^ in mitochondria decreases rapidly, as the distance between organelles and Z-line and transverse tubule in cardiac sarcomeres increases (Sharma et al., 2000; Lu X. et al., 2013). SR-mitochondrial Ca^2+^ transfer seems to occur through direct physical contact in cardiomyocytes. Purified microsomes isolated from rat cardiomyocytes have been shown to be responsive to caffeine stimulation, which is due to the presence of RYR2-containing SR vesicles in these microsomes (Garcia-Perez et al., 2008). This delegate structure allows mitochondria produce ATP closely coupled to RYR2-mediated Ca^2+^ release and the energy demand of the cardiomyocytes (De la Fuente and Sheu, 2019). However, the activity of RYR2 could be enhanced by mitochondrial ROS, leading to aberrant Ca^2+^ leak from the SR and diminished systolic Ca^2+^ transients, contributing to a blunted response to sympathetic stimulation (Cooper et al., 2013). In addition, enhancement of RYR2 activity promotes mitochondrial ROS production in a mitochondrial Ca^2+^-dependent manner, forming a positive feedback process that is detrimental to intracellular Ca^2+^ handling in cardiomyocytes (Hamilton et al., 2020).

MFN2 is the most likely protein involved in the tether between SR and cardiac mitochondria. Under basal conditions, cardiac-specific MFN2 knockout mice show cardiac hypertrophy and moderate diastolic dysfunction, but no systolic dysfunction (Papanicolaou et al., 2011). In contrast, these mice show significant systolic dysfunction under a β-adrenergic stress condition (Papanicolaou et al., 2011). TEM showed that mitochondria were abnormally large and elongated, and the contact between SR and mitochondria was reduced without affecting inter-organelle distance in MFN2 but not MFN1 knockout hearts (Chen et al., 2012). In addition, compared with the control group, MFN2 deficient cardiomyocytes displayed abnormal mitochondrial spatial distribution, low mitochondrial membrane potential and reduced Ca^2+^ uptake (Chen et al., 2012).

In addition, FUN14 domain-containing protein 1 (FUNDC1) is a mitochondrial outer membrane protein involved in maintaining MAM formation by binding with IP3R2. According to the results of TEM, the deletion of FUNDC1 resulted in an 80% reduction in ER and mitochondrial contact (Wu et al., 2017), while MFN2 deletion only resulted in a 30% reduction (Chen et al., 2012). The mutation of FUNDC1 makes it unable to interact with IP3R2, which significantly reduces the contact between ER and mitochondria, similar to the situation after FUNDC1 ablation (Wu et al., 2017). Further studies showed that FUNDC1 inhibited the ubiquitination and degradation of IP3R2 by direct interaction, and improved the stability of IP3R2 (Wu et al., 2017). Mitochondria in the hearts of FUNDC1 knockout mice are larger and more elongated and these mice had significantly lower early and late ventricular filling velocity ratio, decreased ejection fraction, shortening fraction and cardiac output, showing diastolic and systolic dysfunction (Wu et al., 2017).

Mitochondrial dynamics in myocardial tissue is also important for cardiac function. The changes in mitochondrial dynamics can be observed in neonatal rat cardiomyocytes (Kuzmicic et al., 2014), and the fission-fusion cycle is estimated to take nearly 2 weeks in adult cardiomyocytes (Chen et al., 2011). Although the heart appearance of DRP1 knockout mice was normal, the beating rate of isolated cardiomyocytes was lower than that of wild-type mice (Wakabayashi et al., 2009). A recent study demonstrated that decreased DRP-1 expression by mdivi-1 treatment or siRNA knockdown proteolytically cleaved OPA1, and altered the expression of mitochondrial oxidative phosphorylation complex proteins, resulting in defects in mitochondrial respiration, suppressed autophagy and increased mitochondrial ROS production (Aishwarya et al., 2020). Thus, normal mitochondrial fission might be crucial for energy supply in cardiomyocytes. Accordingly, cardiac-specific ablation of DRP1 in mice is lethal, and controlled deletion during adult life results in diminished survival, cardiac hypertrophy, fibrosis and reduced systolic function (Ikeda et al., 2015).

### Vasculature

Compared to cardiac muscle, there are fewer studies on SR-mitochondrial communication in vasculature. Rather than providing energy, mitochondria in ECs are more likely to act as mediators of signal transduction as signaling organelles that control cytosolic Ca^2+^ signaling or modify ROS, as they are reported to be glycolytic and to minimally rely on mitochondria for ATP generation (Wilson et al., 2019). As angiogenesis requires ECs to be in acidic and hypoxic surroundings, a reliance on anaerobic metabolism may enable ECs to form new vessels (Eelen et al., 2018). In ECs, spontaneous ER Ca^2+^ release events and subsequent Ca^2+^ signaling usually occur preferentially at sites of contact between ECs and VSMCs, and this process is maintained and tightly controlled by mitochondrial ATP synthesis (Wilson et al., 2019). MAM formation is increased under hypoxia, which directly induce endothelial mitochondrial damage, leading to elevated ROS production and mitophagy. Disruption of MAMs in ECs attenuates mitochondrial impairment, cell apoptosis, and inflammatory response and increases NO release (Yang et al., 2019). Similarly, increased MAM formation also participates in oxidized low-density lipoprotein (ox-LDL)-induced EC apoptosis, the initial step of atherogenesis. Silencing a tethering protein in MAM, PACS2, inhibits ox-LDL-induced cell apoptosis, as well as concomitant mitochondrial Ca^2+^ elevation, ROS production, and cytochrome c release (Yu et al., 2019). These findings confirm the importance of MAM in regulating endothelial function and participating in related vascular diseases.

In PASMCs, mechanical stress triggers a calcium release by subplasmalemmal RYR1 and is then buffered by mitochondria (Gilbert et al., 2014). The ability of mitochondria to regulate SR-derived Ca^2+^ signal is related to the control of SR luminal Ca^2+^ level, because pharmacological depolarization or MCU inhibition precludes the activation of store-operated Ca^2+^ entry channels, resulting in the decreased VSMC proliferation (Munoz et al., 2011). Also, Ca^2+^/calmodulin-dependent kinase II (CaMKII) in the mitochondrial matrix of VSMCs promotes mitochondrial Ca^2+^ entry by phosphorylating MCU at S92. In a transgenic model of selective mitochondrial CaMKII inhibition in VSMCs, neointimal hyperplasia was significantly reduced after vascular injury (Nguyen et al., 2018). The change of VSMC phenotype is the main cause of many diseases, including hypertension and atherosclerosis, and mitochondrial metabolic regulation is closely related to VSMC differentiation (Owens et al., 2004). SR-mitochondrial communication regulates the oxidative metabolism of VSMCs, because increased Ca^2+^ flow between these organelles is associated with increased mitochondrial activity and more efficient use of glucose (Morales et al., 2014).

Also, little is known about the role of mitochondrial dynamics in ECs and VSMCs compared with that in cardiomyocytes. Some studies have observed changes in mitochondrial morphology that result in the formation of a longer or fragmented network, at least *in vitro*, within a few minutes (Wang et al., 2015; Torres et al., 2016). In most cases, these changes are related to the regulation of cell proliferation. For example, DRP1 activity and mitochondrion fission are required to induce proliferation by mitogenic factors, such as PDGF (Salabei and Hill, 2013; Wang et al., 2015). MFN2 levels are down-regulated when VSMCs switch to proliferative state (Salabei and Hill, 2013). Therefore, the disruption of mitochondrial network seems to be a key step in VSMC proliferation. Recently, TNF-α, a potent pro-inflammatory cytokine, has been discovered to induce mitochondrial fission in ECs via DRP1 (Forrester et al., 2020). Silencing of endothelial DRP1 prevents leukocyte adhesion and proinflammatory proteome induction, suggesting a potential cross-communication between the canonical inflammation pathway and mitochondrial fission (Forrester et al., 2020).

## The Role of Mams in the Development of Cardiovascular Diseases

Compared with the above-mentioned important role of MAM in cardiovascular system physiology, there are few studies on the mechanism of MAM in the occurrence of cardiovascular diseases. So far, studies have shown that MAM proteins are involved in the occurrence of cardiovascular disease, which indirectly reflects the critical role of MAM.

### Ischemia-Reperfusion

The harmful effects of coronary artery disease on myocardium are usually attributed to I/R injury. The subsequent pathological manifestations, from cardiomyocyte death to myocardial injury and cardiac dysfunction, are progressive factors of HF and death (Yellon and Hausenloy, 2007). An important mediator of myocardial I/R injury is mPTP, which is the main driver of cell death (Hausenloy and Yellon, 2003). The abnormal increase of mitochondrial Ca^2+^ leads to the dissipation of mitochondrial membrane potential, mitochondrial swelling and the release of proapoptotic factors, such as cytochrome c, into the cytoplasm, resulting in subsequent pore opening (Ong et al., 2015). On the basis of the results of hypoxia-reoxygenation and I/R, SR-mitochondrial Ca^2+^ transfer is considered to be harmful to cardiac injury. During hypoxia-reoxygenation, the interaction between CypD, a regulatory component of mPTP, and the Ca^2+^ channeling complex (VDAC1-GRP75-IP3R1) increases, resulting in increased mitochondrial Ca^2+^ load and cardiomyocyte death (Paillard et al., 2013). Reduction of CypD-IP3R1 interaction by inhibition of either, using their respective inhibitors NIM811 or 2-aminoethoxydiphenylborate, prevents mitochondrial Ca^2+^ overload and cell death in adult mouse cardiomyocytes (Paillard et al., 2013). Downregulation of CypD or IP3R1 also leads to similar results. In addition, downregulation of MFN2 also prevents mitochondrial Ca^2+^ overload and lethal cell damage by reducing the interaction between CypD and VDAC1-IP3R1 (Paillard et al., 2013). Thus, reducing SR-mitochondrial contact protects against I/R injury by reducing mitochondrial Ca^2+^ overload. Interestingly, ischemic or hypoxic preconditioning, a procedure that protects the myocardium from I/R injury, requires CypD participation to prevent I/R induced cell death, as hypoxic preconditioning reduced cell death in wild-type adult cardiomyocytes, but not in CypD deficient cardiomyocytes exposed to simulated I/R injury (Hausenloy et al., 2010). Similarly, inhibition of CypD by cyclosporin A reduces the beneficial effect of hypoxic preconditioning on I/R (Hausenloy et al., 2010). In addition, treatment of cyclosporin A in adult cardiomyocytes decreased mitochondrial ROS and AKT, the most important mediators of hypoxic preconditioning, and extracellular signal-regulated kinases 1/2 pro-survival activation is reduced in CypD-deficient hearts (Hausenloy et al., 2010). These observations highlight the importance of MAM controlling mPTP and Ca^2+^ channels.

Interacting with the IP3R1-GRP75-VDAC1 complex at the MAM sites, GSK3β is an important regulator of organelle Ca^2+^ transfer in cardiomyocytes (Gomez et al., 2016). Inhibition of GSK3β by SB21 reduces the interactions between the components of this complex and the transfer of Ca^2+^ from SR to mitochondria (Gomez et al., 2016). Similarly, rabbits and mice treated with GSK3β inhibitors (MLS2776 and MLS2778) had smaller infarct size compared with control (Nikolaou et al., 2019). Also, during hypoxia-reoxygenation, SB21 prevented the increase of IP3R1 activity by reducing GSK3β-mediated phosphorylation of IP3R1, and prevented mitochondrial Ca^2+^ overload (Gomez et al., 2016). Consistent with these observations, the use of small interfering RNA targeting VDAC1 can reduce the translocation of GSK3β to mitochondria and prevent the opening of mPTP in response to cellular stress (Tanno et al., 2014). These observations suggest that GSK3β plays an important role in controlling Ca^2+^ flow from SR to mitochondria during I/R. However, loss of GSK-3 in adult cardiac myocytes resulted in induction of mitotic catastrophe, with increased DNA content and multinucleation, leading to apoptosis and severe fatal dilated cardiomyopathy (Zhou et al., 2016).

Cardiomyocyte metabolism is seriously affected by ischemia and hypoxia (Bertero and Maack, 2018). As the main regulator of cell metabolism, mitochondrial dynamics also plays an important role in I/R by regulating the opening of mPTP. As expected, the mitochondrial network is fragmented during ischemia, which is associated with increased cell death (Ong et al., 2010). However, the main function of fission is to produce more mitochondria to meet the energy requirements of myocardial cells during I/R. Inhibition of mitochondrial fission by inactive DRP1 (DRP1K38A) can reduce the sensitivity of HL-1 cells to mPTP opening and reduce cell death induced by I/R (Ong et al., 2010). In addition, inhibitor of mitochondrial fission by mdivi-1 in HL-1 cells and isolated adult cardiomyocytes promotes mitochondrial elongation and decreased cell death after I/R (Ong et al., 2010). Mdivi-1 treatment also reduces the infarct size of the heart in mice with myocardial infarction (Ong et al., 2010). In addition, inhibition of mitochondrial fission by blocking DRP1 function reduces oxygen dependence and increases leak-associated oxygen consumption in cardiomyocytes (Zepeda et al., 2014). This inhibition can produce a protective mitochondrial uncoupling effect and reduce heart injury after I/R (Aldakkak et al., 2008). Similarly, the increased DRP1 activity due to enhanced interaction of filamin A with the GTPase domain of DRP1 also exists in peri-infarct regions characterized by mitochondrial hyperfission (Nishimura et al., 2018). In addition, compared with wild-type mice, mice lacking MFN1 and MFN2 were resistant to the opening of mPTP, which could protect them from acute myocardial I/R injury and reduce the infarct size (Hall et al., 2016). Ischemia also reduces the level of IMM fusion protein OPA1. Although OPA1 overexpression increases mitochondrial fusion in H9c2 myoblasts, it fails to prevent ischemia-induced apoptosis (Chen et al., 2009).

### Diabetic Cardiomyopathy

Diabetic cardiomyopathy is a special form of heart disease, characterized by lipid accumulation in myocardial cells and left ventricular hypertrophy, which leads to systolic dysfunction (Tan et al., 2020). The role of MAMs in diabetes has been studied in detail. For example, in a mouse model of type 2 diabetes mellitus (T2D), disruption of ER-mitochondrial interaction is an early event prior to mitochondrial dysfunction and insulin resistance (Tubbs et al., 2018). Compared with the control group, the number of IP3R-VDAC1 complexes determined by *in situ* proximity ligation assay was significantly reduced in T2D subjects (Thivolet et al., 2017). However, the formation of MAM is increased in diabetic cardiomyopathy. As shown by the quantitative analysis of TEM images, the association between SR and mitochondria increased significantly in the hearts of diabetic Akita mice that carry a single nucleotide substitution in the insulin 2 gene (Wu et al., 2019). Confocal imaging and Pearson correlation coefficient analysis showed that the formation of MAM increased in high glucose (HG) treated neonatal mouse cardiomyocytes (Wu et al., 2019). Similarly, HG significantly increased the expression of FUNDC1 and IP3R2 in neonatal mouse cardiomyocytes (Wu et al., 2019). Compared with non-diabetic donors, FUNDC1 level in heart tissue of diabetic patients was significantly increased (Wu et al., 2019). Moreover, the deletion of FUNDC1 significantly reduced MAM formation in Akita heart (Wu et al., 2019). These results suggest that FUNDC1 is required for diabetic induced cardiac MAM formation. Cardiac-specific FUNDC1 deletion almost completely prevented STZ-induced cardiac abnormalities in diabetic mice, keeping cardiac function at normal values (Wu et al., 2019). It can be speculated that the increase of MAM in high glucose environment may cause mitochondrial calcium overload and damage mitochondrial function, which is the key determinant of HF. Metformin, a famous antidiabetic drug, activates AMP-activated protein kinase (AMPK), improves cardiac function by restoring mitochondrial and cardiac ultrastructure in mice (Xie et al., 2011), and reduces the incidence of myocardial infarction in diabetic patients (Griffin et al., 2017). Studies have shown that the inactivation of AMPK leads to diabetic cardiomyopathy by increasing the MAMs associated with FUNDC1 (Wu et al., 2019). In contrast, we recently observed a negative role of AMPK on MAM formation by reducing the expression of FUNDC1 (Wei et al., 2020). Also, under energy stress, considerable amounts of AMPK translocate from cytosol to the MAM and the mitochondrion as mitochondrial fission occurs, where they interact directly with MFN2 to initiate autophagy (Hu Y. et al., 2020). These findings suggest that the cardiovascular benefits of metformin may depend on its regulation of MAM formation.

### Heart Failure (HF)

Coronary artery disease, as well as other cardiovascular diseases, initially leads to compensatory myocardial hypertrophy, which, if worsened, can lead to HF. In the process of cardiac hypertrophy and its transition to HF, changes in SR- mitochondrial contact have been observed (Gutierrez et al., 2014; Santulli et al., 2015). In the norepinephrine-induced hypertrophic environment, the distance between SR and mitochondria in cardiomyocytes is increased, which reduces the Ca^2+^ re-uptake of mitochondria (Gutierrez et al., 2014). This may be a compensatory or adaptive mechanism to buffer the increased SR Ca^2+^ leakage during HF. However, it results in the decrease of mitochondrial oxidative activity, which forced the metabolism of cardiomyocytes into glycolysis, thus promoting the occurrence of hypertrophy (Bertero and Maack, 2018). In addition, SR-mitochondrial communication deficiency and low efficiency of Ca^2+^ exchange may be the prerequisite for pathological myocardial hypertrophy in aged mice (Fernandez-Sanz et al., 2014). Similarly, blocking SR-mitochondrial Ca^2+^ transfer by cardiac-specific knockout of RYR2 results in spontaneous myocardial hypertrophy and fibrous hyperplasia in mice (Bround et al., 2013). Therefore, mitochondrial Ca^2+^ maladjustment seems to be an obvious feature of HF. However, there is no conclusive data on the changes of mitochondrial Ca^2+^ level. Some reports have shown that significant perturbations of cytosolic cation levels have been observed in HF, including an increase in Na^+^ levels (Shimizu and Minamino, 2016), which contributes to the outflow of mitochondrial Ca^2+^ through the mitochondrial NCLX and reduces the mitochondrial bioenergetic responses (Maack et al., 2006). In the guinea pig HF model, the increase of Na^+^ in cytoplasm promoted the production of mitochondrial H_2_O_2_ (Kohlhaas et al., 2010). Interestingly, a compound blocking NCLX (CGP-37157) enhanced mitochondrial Ca^2+^ accumulation, reduced H_2_O_2_ production, and restored mitochondrial energy supply (Liu and O’Rourke, 2008; Kohlhaas et al., 2010). Contrary to the above results, mitochondrial Ca^2+^ overload caused by SR Ca^2+^ leakage through RYR2 channel was detected in the mouse model of posy-myocardial infarction, leading to changes in normal mitochondrial function and a harmful increase in mitochondrial Ca^2+^ levels (Santulli et al., 2015). The researchers suggested that there was a positive feedback loop between SR Ca^2+^ leakage and mitochondrial ROS production, which leads to RYR2 leakage and intracellular Ca^2+^ increase (Santulli et al., 2015). In line with this view, blocking CaSR reduces the intercellular Ca^2+^ transfer, mitochondrial Ca^2+^ overload and apoptosis (Lu F. H. et al., 2013).

Inadaptable cardiac hypertrophy, which leads to HF, can produce a variety of functional disorders, including changes in mitochondrial dynamics. Decreased MFN2 levels were observed in both *in vitro* and *in vivo* models, such as spontaneously hypertensive rats and hypertrophy caused by pressure overload induced by transverse aortic contraction (Fang et al., 2007). In addition, decreased OPA1 levels were associated with mitochondrial network fragmentation in rat and human HF models (Chen et al., 2009), and decreased MFN1 and MFN2 levels associated with mitochondrial network changes were detected in guinea pig HF models (Goh et al., 2016). Norepinephrine can induce cardiomyocyte hypertrophy and mitochondrial fission by regulating DRP1 function (Pennanen et al., 2014). DRP1 acetylation increases its activity and mitochondrial translocation, resulting in cardiomyocyte hypertrophy and dysfunction in response to excessive lipid supply (Hu Q. et al., 2020). Overexpression of inactive DRP1 (DRP1K38A) in cultured neonatal rat cardiomyocytes prevented mitochondrial network damage and noradrenaline-induced hypertrophy (Pennanen et al., 2014). It should be noted that in the absence of other external stimuli, reducing MFN2 levels is sufficient to induce hypertrophic cardiomyocyte growth (Pennanen et al., 2014). Consistent with these results, moderate myocardial hypertrophy and mild functional deterioration were observed in cardiac-specific MFN2 deficient mice (Papanicolaou et al., 2011). On the other hand, down-regulation of OPA1 increases the response of myocardium to mechanical stress. Higher levels of cardiac hypertrophy associated with changes in ventricular function were detected in OPA1^+/–^ mice exposed to transverse aortic contraction compared to wild-type mice (Piquereau et al., 2012). Cardiac-specific deletion of ATP-dependent zinc metalloproteinase YME1L1 impairs mitochondrial morphology and leads to progressive dilated cardiomyopathy by increasing OPA1 degradation (Wai et al., 2015). Therefore, maintaining the balance between mitochondrial fusion and fission seems to be the key to maintaining normal cardiac function. Treatments that regulate mitochondrial dynamics can be used to prevent cardiac hypertrophy and HF. For example, in a mouse model of pressure overload induced by transverse aortic contraction, mdivi-1 treatment can reduce cardiac fibrosis and left ventricular dysfunction (Givvimani et al., 2012). However, a 2016 report showed that DRP1-dependent mitophagy has a protective effect on HF induced by pressure overload (Shirakabe et al., 2016), so mdivi-1 may have a harmful effect on the later stage of HF.

Endoplasmic reticulum stress involving PERK and eIF2α-ATF4-CHOP signaling has recently been considered as a critical step for development of cardiac hypertrophy and HF. As a member of ER reticulon family that define the tubular morphology of the ER, NOGO B acts as a negative regulator of ER–mitochondria contacts (Sutendra et al., 2011). Inhibition of NOGO B promotes cardiomyocyte hypertrophy and cardiac fibroblast activation by activating the PERK/ATF4 signaling pathway and ATF6 branches of ER stress pathways (Li et al., 2018).

### Pulmonary Arterial Hypertension

Pulmonary arterial hypertension (PAH) is a progressive and fatal disease characterized by the gradual increase of pulmonary vascular resistance and pulmonary arterial pressure, which eventually leads to right ventricular dysfunction and death (Galie et al., 2016). The long-term survival rate of PAH is depressing. The survival rate of patients with PAH decreases significantly after symptoms appear, and lung transplantation is the only choice for patients with advanced disease (Galie et al., 2016). PAH is characterized by vascular remodeling caused by phenotypic changes in PASMCs. In PAH, PASMCs with quiescent contractile phenotype transit to a highly proliferative phenotype and resistance to apoptosis, resulting in occlusion of small pulmonary vessels (Galie et al., 2016). Although the etiology of PAH is diverse and often multifactorial, phenotypic transformation, proliferation, hypertrophy and other cytological behaviors of PASMC are related to the changes of metabolic patterns (Paulin and Michelakis, 2014; Sutendra and Michelakis, 2014). Specifically, PASMC converts from ATP production from mitochondrial oxidative substrates to relying primarily on cytoplasmic glycolysis for energy, similar to the Warburg effect described in cancer cells (Cottrill and Chan, 2013; Sutendra and Michelakis, 2014). At the same time, the inhibition of mitochondrial oxidation leads to the decrease of mitochondrial membrane hyperpolarization and ROS, which increases the threshold of mPTP opening and apoptosis in PASMC, and the metabolites previously oxidized in mitochondria can now be used as precursors to synthesize macromolecules needed for cell growth and proliferation (Cottrill and Chan, 2013; Sutendra and Michelakis, 2014). The inhibition of mitochondrial oxidation in PAH may be the consequence of altered functional contact between SR and mitochondria, which leads to the decrease of mitochondrial Ca^2+^ concentration and Ca^2+^-dependent dehydrogenase activity. As the gatekeeper of complete oxidation of glucose in mitochondria, PDH complex converts pyruvate derived from glycolysis into mitochondrial Ac-CoA. Therefore, PDH is a key factor of metabolic transfer observed in PASMCs, and its activity is inhibited by phosphorylation (Sutendra and Michelakis, 2014). Mitochondrial Ca^2+^ regulates the phosphorylation of PDH by activating PDH phosphatase (Denton et al., 1972) and inhibiting PDH kinase (PDK) (Cooper et al., 1974), thus enhancing PDH activity and facilitating glucose complete oxidation. Activation of PDH with dichloroacetic acid (PDK inhibitor) can prevent hypoxia-induced metabolic and phenotypic changes in PASMCs (McMurtry et al., 2004; Sutendra and Michelakis, 2014). On the other hand, mitochondrial UCP2 acts as a selective modulator of MCU-dependent mitochondrial Ca^2+^ inward current (Bondarenko et al., 2015), while UCP2 knockout in PASMCs reduces mitochondrial Ca^2+^ level and the activity of Ca^2+^-dependent enzymes, simulating the effect of hypoxia (Dromparis et al., 2013b). In addition, UCP2-deficient mice produce spontaneous PAH, which highlights the relevance of this mechanism in the development of the disease (Dromparis et al., 2013b). Similarly, we also noticed that UCP2 deficiency was associated with increased mitochondrial ROS generation and reduced NO production in the endothelium (Xiong et al., 2016), which might also play a role in vascular dysfunction.

The common characteristics of several PAH related diseases, such as hypoxia, viral infection and inflammation, are the causes of SR stress (Sutendra and Michelakis, 2014). The activation of ATF6α axis, a cAMP-dependent transcription factor of SR stress response, leads to the increase of NOGO B expression in SR (Sutendra et al., 2011), which leads to the structural change of SR, and increases the distance between the organelle and mitochondria, leading to the damage of mitochondrial function (Sutendra et al., 2011; Sutendra and Michelakis, 2014). According to the mechanism of PAH, mice lacking NOGO B are resistant to PAH induced by chronic hypoxia (Sutendra et al., 2011). In addition, the use of small-molecular chemical chaperones prevents and reverses the established PAH and its related cell phenotypes in two rodent disease models by reducing SR stress (Dromparis et al., 2013a).

Another aspect of mitochondrial function associated with phenotypic changes observed in PASMCs is mitochondrial dynamics. As reported in other cell types, the increased proliferation of PASMCs in the pathogenesis of PAH is coordinated with the disruption of mitochondrial network in M phase, so that the distribution of mitochondria in daughter cells is equal (Taguchi et al., 2007; Ryan et al., 2015). In fact, PASMCs isolated from PAH patients showed that mitochondrial fragmentation was associated with decreased expression of the fusion protein MFN2 and increased levels of the fission protein DRP1 (Taguchi et al., 2007; Ryan et al., 2015). MiR-34a-3p-mediated epigenetic upregulation of DRP1 adapter proteins MiD49 and MiD51 increases mitotic fission, which drives pathological proliferation and apoptosis resistance in PAH (Chen et al., 2018). It should be noted that the phenotypic changes of PASMCs can be prevented by controlling mitochondrial dynamics by mdivi-1 treatment or MFN2 overexpression. These strategies produce cell cycle arrest in PASMCs of PAH patients *in vitro*, reverse the established PAH *in vivo*, restore pulmonary artery remodeling, reduce pulmonary vascular resistance and right ventricular hypertrophy, and improve the motor ability of rodent models (Marsboom et al., 2012; Ryan et al., 2013). In addition, MFN2 not only plays a role in mitochondrial fusion, but also participates in the binding and communication between SR and mitochondria. In PAH patients and mouse models, the decrease of MFN2 level may also be related to the increased distance between the two organelles (Ryan et al., 2013).

### Systemic Cardiovascular Diseases

In addition to the respiratory system, the phenotypic changes of VSMCs also play a role in the development of different diseases, such as hypertension and atherosclerosis (Chiong et al., 2013). Although there is indirect evidence to support the role of SR-mitochondrial communication in these diseases, some studies have shown that this communication plays a role in the pathogenesis of these diseases. Using high-resolution confocal microscopy and proximity ligation assays, Moulis et al. (2019) found an increase in MAM contacts in VSMCs upon stimulation with atherogenic lipids. Unlike what has been previously described in ECs, the disruption of MAM contacts by PACS-2 knockdown facilitated VSMC apoptosis, an initial step for atherogenesis and plaque rupture, by inhibiting mitophagosome formation and mitophagy (Moulis et al., 2019). Most studies have focused on the role of MFN2 in the transition of VSMCs from contractile and resting phenotypes to hyperproliferative and migratory phenotypes. In this regard, VSMCs from spontaneously hypertensive rats or balloon-injured arteries showed higher proliferation rate and lower level of MFN2 (Wang et al., 2015; Torres et al., 2016). On the contrary, MFN2 overexpression inhibited the proliferation of these cells, neointima formation and carotid restenosis induced by balloon injury in rat carotid arteries (Chen et al., 2004; Guo X. et al., 2007; Guo Y. H. et al., 2007). In addition, in apolipoprotein E (ApoE)-deficient mice, the progression of carotid atherosclerosis is accompanied by a decrease in MFN2 levels (Chen et al., 2004), and overexpression of MFN2 inhibits oxLDL-induced VSMC proliferation during atherogenesis (Guo Y. H. et al., 2007). Although these findings do not indicate the specific role of SR-mitochondrial contact in vascular pathology, the correlation between MFN2 level, VSMC proliferation and SR-mitochondrial contact has been observed in view of the other functions of MFN2 besides organelle tethering. In rat aortic VSMCs, MFN2 level increased in *G*_0_/*G*_1_ phase, mitochondrial elongation and MAMs increased (Li et al., 2015). Similarly, the overexpression of MFN2 was associated with *G*_0_/*G*_1_ phase arrest and increased number of renal tubular mitochondria and SR-mitochondrial contact sites. On the contrary, MFN2 gene knockout was associated with the increase of S phase cells, the disruption of mitochondrial network and the decrease of SR-mitochondrial contact sites (Li et al., 2015).

Although there is no direct evidence for the role of SR-mitochondrial contact in the pathogenesis of systemic vascular diseases, there is more data on the role of mitochondrial dynamics in this area. During the phenotypic changes of VSMCs, the decrease of MFN2 expression may be related to the mitochondrial network disruption observed in proliferative cells. In this regard, PDGF, a known inducer of VSMC phenotype changes, has been shown to increase mitochondrial fission and VSMC proliferation and migration (Salabei and Hill, 2013; Wang et al., 2015). High glucose concentration in the culture media, is an experimental condition that mimics the high glucose levels present in the blood of people affected by diabetes, can also induce high proliferation phenotype of VSMCs cultured *in vitro*, indicating the development of diabetic vascular complications (Maimaitijiang et al., 2016). DRP1 has been found enriched in calcified regions of human carotid arteries, and mice heterozygous for DRP1 deletion are resisted to vascular calcification in an atherosclerosis model (Rogers et al., 2017). It should be noted that inhibition of mitochondrial fission by mdivi-1 or by expression of DRP1K38A can prevent VSMCs from over proliferation at high glucose level (Maimaitijiang et al., 2016), AngII infusion (Cooper et al., 2020) or PDGF stimulation (Salabei and Hill, 2013; Wang et al., 2015). In addition, DRP1K38A transgene in mice displayed a protective effect on intimal vascular proliferation after vascular injury (Wang et al., 2015).

## Conclusion

**FIGURE 2:**
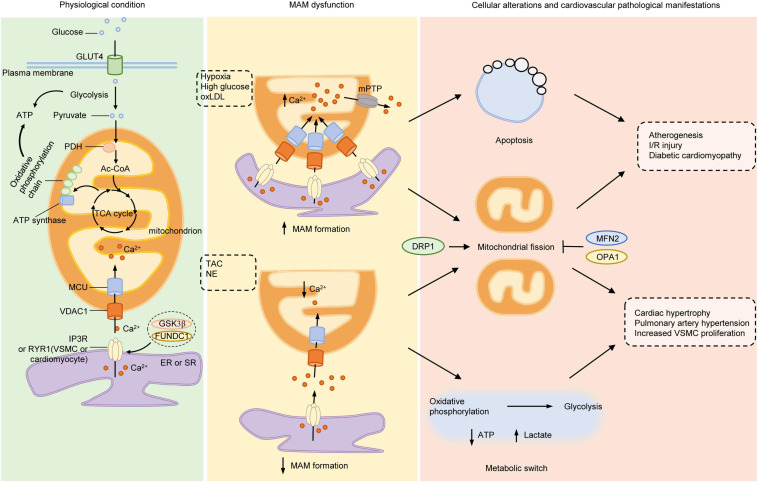
Mitochondria-associated ER membranes and pathogenesis of cardiovascular diseases. Under normal conditions, glucose is taken up into cytoplasm via GLUT4, where it is divided into two molecules of pyruvate through glycolysis. PDH converts pyruvate derived from glycolysis into mitochondrial Ac-CoA, which enters into TCA cycle to generate substrates required for electron transfer of oxidative phosphorylation chain located on IMM. Both glycolysis and mitochondrial oxidative phosphorylation produce ATP but the efficiency of mitochondria is much higher than glycolysis. Ca^2+^ transferred from ER to mitochondria via IP3R (or RYR in VSMCs and cardiomyocytes)/VDAC1 complex acts as stimulants of main enzymes in TCA cycle to enhance the ability of mitochondrial bioenergetics. Under pathological stimulants, such as hypoxia, high glucose or ox-LDL, the formation of MAMs increases and lead to excessive mitochondrial Ca^2+^ uptake, which opens the mPTP to initiate apoptosis and mitochondrial fragmentation, the early steps of atherogenesis, I/R injury and diabetic cardiomyocyte damage. On the other hand, under TAC or NE stimulation, the formation of MAMs is reduced, with increased gap between ER and mitochondria, resulting in lowered mitochondrial Ca^2+^ level and elevated cytosolic Ca^2+^. The insufficient mitochondrial Ca^2+^ lowers the activity of oxidative phosphorylation, initiates metabolic switch to glycolysis to generate ATP, resulting in increased lactate production and mitochondrial fission. Inhibition of mitochondrial energy production evokes phenotype switch of VSMCs or cardiomyocytes from contractile to synthetic, a major step for developing hypertrophy-associated diseases, such as HF, PAH, and systemic vascular diseases. Mitochondrial dynamics participates in both processes as either inhibition of DRP1 or activation of MFN2 or OPA1 exerts beneficial effects on cardiovascular diseases. Ac-CoA, acetyl-coenzyme A; DRP1 dynamin-related protein 1; ER, endoplasmic reticulum; FUNDC1, FUN14 domain-containing protein 1; GLUT4, glucose transporter type 4; GSK3β, glycogen synthase kinase 3β; IMM, inner mitochondrial membrane; IP3R, inositol-1,4,5-triphosphate receptor; I/R, ischemia-reperfusion; MAMs, mitochondria-associated ER membranes; MCU, mitochondrial calcium uniporter; MFN2, mitofusin 2; mPTP, mitochondrial permeability transition pore; NE, norepinephrine; OPA1, optic atrophy protein 1; ox-LDL, oxidative low-density lipoprotein; PDH, pyruvate dehydrogenase complex; RYR, ryanodine receptor; TAC, thoracic aortic constriction; TCA, tricarboxylic acid; VDAC1, voltage-dependent anion-selective channel protein 1; VSMC, vascular smooth muscle cell.

As MAM connects two important organelles, ER and mitochondria, the formation, structure and function of MAM display significant regulatory roles in cellular behaviors associated with ER and mitochondria. This review summarizes the function and mechanism of MAM in regulating cell behavior, and its importance in cardiovascular physiology and pathophysiology from three aspects. First, the Ca^2+^ transfer from ER to mitochondria mediated by MAM affects the energy metabolic patterns of cardiovascular cells, which are related to cardiomyocyte hypertrophy, VSMC phenotype transition and proliferation. Second, the Ca^2+^ transfer of MAM not only affects the Ca^2+^ levels of mitochondria and ER itself, but also affects the local cytosolic Ca^2+^ concentration which might induce subsequent pro-hypertrophic Ca^2+^ signaling and contractile response. Third, MAM not only triggers the cellular behaviors related to ER and mitochondria by modulation lipid and Ca^2+^ transfer, such as ER stress and apoptosis caused by mitochondrial Ca^2+^ overload, but also acts as a platform where inflammasome and mitochondrial fission occurs by recruiting key signal molecules and effector proteins. Thus, the interaction between these organelles is an important factor in the pathophysiology of cardiovascular system (Figure 2). However, the role of ER-mitochondrial communication in cardiovascular diseases has not been paid enough attention, especially in the blood vessels with less mitochondrial content. Although many molecules involved in the regulation of MAM function are summarized in this paper, their functions in cardiovascular system have not been fully studied.

At present, there are two major obstacles in the way of identifying the role of MAM in the cardiovascular system. On the one hand, cardiomyocytes mainly rely on mitochondria for energy supply, whereas glycolysis is predominant in vascular ECs, and VSMCs stand between the two. Obviously, the effects of MAM formation in these cell types are not identical, sometimes even opposite. Therefore, it is very important to explore the role of MAM in a cell-specific manner, which also raises a question for us that how to regulate the formation and function of MAM according to different cell types, so as to develop new treatment methods for cardiovascular diseases. Regulating communication between these organelles, on the other hand, is a double-edged sword. The increase of ER-mitochondrial proximity increases mitochondrial Ca^2+^ uptake, thus activated ATP synthesis, while mitochondrial Ca^2+^ overload triggered the opening of mPTP, resulting in mitochondrial swelling and pro-apoptotic factors released to the cytoplasm. Thus, the concentration of mitochondrial Ca^2+^ needs to be appropriately controlled. Therefore, the precise regulation of MAM formation and function might be a promising way to develop more specific treatment strategies and selectively restrain the progression of cardiovascular diseases.

## Author Contributions

PG, ZY, and ZZ designed the scope of the review. PG performed the document searching and wrote the manuscript. PG prepared the figures. All authors contributed to the article and approved the submitted version.

## Conflict of Interest

The authors declare that the research was conducted in the absence of any commercial or financial relationships that could be construed as a potential conflict of interest.

## Abbreviations

Ac-CoA: acetyl-coenzyme A
AKT: serine/threonine kinase
AMPK: adenosine 5 ′ -monophosphate (AMP)-activated protein kinase
ASC: apoptosis-associated speck-like protein
ATF6: activating transcription factor 6
ATG14L: autophagy-related 14-like
BCL-XL: B cell lymphoma extra-large
Ca^2+^: calcium
CaMKII: Ca^2+^/calmodulin-dependent kinase II
cFLIPL: FADD-like apoptosis regulators
CypD: peptidyl-prolyl *cis*–*trans* isomerase D
DRP1: dynamin-related protein 1
EC: endothelial cell
ER: endoplasmic reticulum
FACL4: fatty acid CoA ligase 4
Fis1: fission 1 protein
FUNDC1: FUN14 domain-containing protein 1
GLUT4: glucose transporter type 4
GRP75: chaperone 75 kDa glucose-regulated protein
GSK3β: glycogen synthase kinase 3 β
HF: heart failure
IMM: inner mitochondrial membrane
IL-1β: interleukin 1 β
IP3R: inositol-1,4,5-triphosphate receptor
I/R: ischemia-reperfusion
IRE1: inositol-requiring enzyme 1
MAM: mitochondria-associated ER membrane
MCU: mitochondrial calcium uniporter
MFN2: mitofusin 2
Mff: mitochondrion fission factor
mPTP: mitochondrial permeability transition pore
mTORC2: mammalian target of rapamycin complex 2
NE: norepinephrine
NCLX: Na^+^/Ca^2+^ exchanger 1
NO: nitric oxide
NOGO B: neurite outgrowth inhibitor B
NLRP3: pyrin domain-containing 3 protein
OMM: outer mitochondrial membrane
OPA1: optic atrophy protein 1
ORP5: oxysterol-binding protein-related protein 5
ox-LDL: oxidative low-density lipoprotein
PACS2: phosphofurin acidic cluster sorting protein 2
PAH: pulmonary arterial hypertension
PASMC: pulmonary artery smooth muscle cell
PDGF: platelet-derived growth factor
PDH: pyruvate dehydrogenase complex
PDK: PDH kinase
PE: phosphatidylethanolamine
PEMT2: phosphatidyl ethanolamine methyltransferase 2
PERK: protein kinase-like ER kinase
PML: promyelocytic leukemia protein
PS: phosphatidylserine
PSS: PS synthase
PTEN: phosphatase and tensin homolog
RMDB3: regulator of microtubule dynamics 3
ROS: reactive oxygen species
RYR: ryanodine receptor
SERCA: sarco/endoplasmic reticulum Ca^2+^-ATPase
SR: sarcoplasmic reticulum
TAC: thoracic aortic constriction
TCA: tricarboxylic acid
TEM: transmission electron microscopy
TNF-α: tumor necrosis factor- α
TRPM8: transient receptor potential melastatine 8
TXNIP: thioredoxin interacting protein
T2D: type 2 diabetes mellitus
UCP2: uncoupling protein 2
UPR: unfolded protein reaction
VAMP: vesicle associated membrane protein
VAPB: VAMP-associated protein B
VDAC1: voltage-dependent anion-selective channel protein 1
VSMC: vascular smooth muscle cell
YME1L1: YME1 Like 1 ATPase.
